# Out-of-Pocket and Informal Payment Before and After the Health Transformation Plan in Iran: Evidence from Hospitals Located in Kurdistan, Iran

**DOI:** 10.15171/ijhpm.2017.16

**Published:** 2017-02-11

**Authors:** Bakhtiar Piroozi, Arash Rashidian, Ghobad Moradi, Amirhossein Takian, Hooman Ghasri, Tayyeb Ghadimi

**Affiliations:** ^1^ Department of Health Services Management and Economics, School of Public Health, Tehran University of Medical Sciences, Tehran, Iran.; ^2^ Knowledge Utilization Research Center, Tehran University of Medical Sciences, Tehran, Iran.; ^3^ Social Determinants of Health Research Center, Kurdistan University of Medical Sciences, Sanandaj, Iran.; ^4^ Department of Global Health and Public Policy, School of Public Health, Tehran University of Medical Sciences, Tehran, Iran.; ^5^ National Academy of Medical Sciences, Tehran, Iran.; ^6^ College of Health and Life Sciences, Brunel University London, London, UK.; ^7^ Deputy of Treatment, Kurdistan University of Medical Sciences, Sanandaj, Iran.; ^8^ Department of Surgery, Faculty of Medicine, Kurdistan University of Medical Sciences, Sanandaj, Iran.

**Keywords:** Informal Payment, Health System Reform, Out-of-Pocket (OOP) Payment, Health Expenditure

## Abstract

**Background:** One of the objectives of the health transformation plan (HTP) in Iran is to reduce out-of-pocket (OOP) payments for inpatient services and eradicate informal payments. The HTP has three phases: the first phase (launched in May 5, 2014) is focused on reducing OOP payments for inpatient services; the second phase (launched in May 22, 2014) is focused on primary healthcare (PHC) and the third phase utilizes an updated relative value units for health services (launched in September 29, 2014) and is focused on the elimination of informal payments. This aim of this study was to determine the OOP payments and the frequency of informal cash payments to physicians for inpatient services before and after the HTP in Kurdistan province, Iran.

**Methods:** This quasi-experimental study used multistage sampling method to select and evaluate 265 patients ischarged from hospitals in Kurdistan province. The study covered 3 phases (before the HTP, after the first, and third phases of the HTP). Part of the data was collected using a hospital information system form and the rest were collected using a questionnaire. Data were analyzed using Fisher exact test, logistic regression, and independent samples t test.

**Results:** The mean OOP payments before the HTP and after the first and third phases, respectively, were US$59.4, US$17.6, and US$14.3 in hospital affiliated to the Ministry of Health and Medical Education (MoHME), US$39.6, US$33.7, and US$13.7 in hospitals affiliated to Social Security Organization (SSO), and US$153.3, US$188.7, and US$66.4 in private hospitals. In hospitals affiliated to SSO and MoHME there was a significant difference between the mean OOP payments before the HTP and after the third phase (P<.05). The percentage of informal payments to physicians in hospitals affiliated to MoHME, SSO, and private sector, respectively, were 4.5%, 8.1%, and 12.5% before the HTP, and 0.0%, 7.1%, and 10.0% after the first phase. Contrary to the time before the HTP, no informal payment was reported after the third phase.

**Conclusion:** It seems that the implementation of the HTP has reduced the OOP payments for inpatient services and eradicated informal payments to physician in Kurdistan province.

## Background

The share of out-of-pocket (OOP) health payments in total health expenditures and the subsequent catastrophic health expenditures (CHEs) are the 2 important factors that should be taken into account while planning and designing health policies.^1,2^ Too much reliance on OOP payments prevents countries from reaching universal health coverage (UHC).^3-6^ Heavy reliance on OOP payments deprives millions of people from receiving healthcare services when they are in need of such services.^3^ OOP payment is the most unfair and inefficient method of financing in the health sector and could lead to an increase in poverty.^4^ Globally, about 150 million people are faced with CHE because of OOP payments to the health sector, of whom about 100 million people are pushed below the poverty line.^7^ However, OOP payments are common in most of developing countries for different reasons such as the lack or inefficiency of health insurance system, low share of health budget from the total budget of governments, and low government budgets. As a consequence, these types of payments are the main source of financing of the health sector for 33 countries. In such countries more than 50% of total healthcare expenditures are financed via OOP payments.^3,8^ The distribution of the burden of OOP payments among different income groups is regressive because low-income groups, compared with the high-income groups, allocate a greater share of their income to pay for the health services expenditures.^9,10^ Utilization of prepayment schemes, such as health insurance, can reduce OOP payments and decrease the risk of CHEs.^3,11,12^ OOP payments are the sum of all payments by patients for outpatient and inpatient health services which are not reimbursed by patient’s health insurance company. Moreover, OOP payments for inpatient services include all direct payments by patients for receiving hospital inpatient care services.^3,13,14^

Generally, there are 2 types of OOP payments for inpatient services in Iran, including (1) formal payments in and out of hospitals at the point of receiving hospital care services and (2) informal payments to healthcare providers.^13,15,16^ In Iran, informal patient payment is defined as a cash or in-kind supplement to the official payment.^15,16^ Informal payments usually occur outside the official payment channels.^15,17,18^ Because of 3 characteristics of information asymmetry, uncertainty, and the large number of actors, the health sector is at an increased risk of corruption.^19^

The literature suggests different reasons for receiving and giving informal patient payments in the health systems, including the followings: low government investment in the health sector, unrealistic tariffs, lack of transparency and oversight in the health sector, monopoly, defects of insurance organizations, poor management, low salaries of health service providers, poor quality of health services, and cultural characteristics of a community.^20-22^ These payments can also lead to many negative consequences including the followings: lack of poor people’s access to health services, increased inequality in the utilization of health services, poor people’s unwillingness or delay in the utilization of services, promotion of useless and non-essential services, demolishing the social status of physicians, and harms to the physician-patient relationship.^21-25^

Informal patient payment is common and prevalent in many low- and middle-income countries and has become a challenge for health policy-makers.^17,26^ The results of a study on 29 developing countries with transitional economies showed that the frequency of informal payments among users of healthcare services ranged from 3.0% in Peru to 96.0% in Pakistan.^20^ According to a study conducted in Greece in 2008, 36.0% of patients admitted to hospitals affiliated to the reported at least one case of informal payments to physicians. Of those, 42.0% of patients had an informal payment due to the fear of receiving sub-standard care while 20.0% paid informally due to physicians request.^27^

To afford OOP payments, poor households usually use different methods such as selling assets and borrowing from relatives and banks.^20,28^ Recently, there has been increasing efforts and cooperation among policy-makers, planners, and donors in the health sector to combat corruption in the health sector.^18^ While designing strategies to fight informal patient payments and other forms of corruption, it is necessary to consider the texture and specific problems of the health sector.^18,29^ According to the results of studies in various countries, the increased awareness of citizens about informal payments and the methods of reporting such practices can have positive effects on reducing informal payments and corruption in the health sector.^29,30^ Governments around the world initiated the process of health sector reforms to protect households from the risk of impoverishment due to OOP expenses for healthcare services.^31^ Health sector reform in the Africa is focused on new methods of financing, organizing, managing, and implementing social insurance programs.^32,33^ The transition economies of Eastern Europe have proposed many new social insurance schemes.^34^ Some countries have had some achievements to solve this problem.^31,35^ Since 2003, Turkey has brought about a series of reforms in the health sector which are managed under the health transformation program (HTP); this program is aimed at achieving UHC, reducing OOP payment, decreasing health inequalities in co-financing healthcare expenditures, increasing people’s access to health services, and improving health outcomes.^36^

### An Overview of Iran Healthcare System

In Iran, primary healthcare (PHC) services are financed and provided by the government across the country through a widespread PHC network. The second and third level services are provided by the public, semi-public, and private sectors. Iran has 3 main insurance organizations including the Iranian Health Insurance Organization (IHIO), Social Security Organization (SSO), and Armed Forces Insurance Organization (AFIO). IHIO and SSO have multiple insurance schemes, which cover both compulsory and optional insurances, while the AFIO schemes are only compulsory. In recent years, despite growing developments in the healthcare system in Iran, the equitable financing has been one of the major challenges faced by the country’s health system.^2^ Some of the problems in this area are as follows:

- High share of OOP payments in the total healthcare expenditures: The fifth economic, social, and cultural development plan of Iran has emphasized on reducing OOP payments and CHE to less than 30.0% and 1.0%, respectively,^37^ however, in recent years the share of OOP payments has raised and accounted for over 50.0% of the total healthcare expenditures.^38^ Moreover, according to the studies conducted in different parts of the country, the percentage of households exposed to CHE has been between 8.3%-22.0%.^2,13,39,40^
- Low share of the health budget in the total budget of the government: in recent years before the HTP (May 2014), the governmental budget allocated to the health sector has been less than 8.0% of the total budget of the government.^37,41^
- Inefficiency of national insurance system in three dimensions of the total population under the coverage (Breadth), services under the coverage (Depth), and the level of financial support for services (Height): According to a report by the National Institutes of Health Research, 17.0% of households were not under the coverage of any health insurance system in 2010.^42^ In addition, according to a report by the Iranian National Health Accounts in 2008, the three main insurance organizations in Iran, ie, IHIO, SSO and AFIO, respectively, incurred 6.6%, 10.9%, and 1.5% of the total healthcare expenditures, which indicated the inefficiency of insurers in covering the costs of services. Thus, the largest part of the healthcare expenditures in Iran is paid by the households.^37,41^
- The prevalence of informal payments in the health sector: despite the illegal nature of informal payments, this type of payments has been reported in the health sector in recent years. According to a study in 2013, 11.2% of physicians had received informal payments from hospitalized patients. The mean informal payments received by physicians with different specialization were as follows: US$150 by gynaecology and obstetrics specialists, US$286.9 by general surgery specialists, US$250 by internal medicine and neurology specialist, US$31.3 by nephrologists, US$812.5 by orthopaedic specialist, US$450.6 by otolaryngology specialists.^15^ According to some other studies in Iran, from the perspective of practitioners, policy-makers, and patients, the major causes of informal payments are: cultural factors, factors related to the quality of services, inefficiency of the health system, unrealistic tariffs, direct financial relationship between physicians and patients, and financial weakness of insurance agencies.^21,22^

As mentioned above, in recent years, there have been many driving forces to reform the health sector in Iran, but the greatest obstacle facing the reform is the lack of political support.^43^ Nevertheless, the eleventh presidential cabinet has paid a special attention to the health sector. Political and financial supports have been provided by the government and parliament to reform the health sector. Accordingly, the Ministry of Health and Medical Education (MoHME) has started the HTP since May 5, 2014, to conduct a series of reforms to increase equity and reach UHC in Iran.^44^

### What Is the Health Transformation Plan in Iran?

The HTP includes a series of reforms that has been implementing in three phases since May 5, 2014 in Iran. The first phase of the HTP focused on hospitals affiliated to MoHME, the second phase with a focus on PHC started on May 22, 2014; this phase was not covered by our study as this phase was not expected to affect inpatients’ payments, at least in a short run. The third phase, which updated tariffs of health services, began on September 29, 2014 and it was implemented in all parts of the health system. In recent years, the majority of physicians have complained about unrealistic tariffs as the main reason for receiving informal payments. With the implementation of the third phase of the HTP, universities of medical sciences began a strict monitoring on the health service tariffs, so that to avoid requests for extra payments. Paying fines, calling off the contracts by insurance companies, firing the presidents of public hospitals, lowering the hospital accreditation grade, and reimbursing patients’ payments are among the punishments for those who receive informal payments.^45,46^ Different intervention and main objectives of the HTP are listed in Table 1.

**Table 1:** Interventions and objectives of Iran’s HTP

**Phase**	**Interventions**	**Objectives**
First	• Providing basic health insurance free of charge by the Iran Health Insurance Organization for all Iranians without any health insurance coverage and covering some of the services that were not previously covered by the service packages of basic insurance systems. • Reducing the share of OOP payments to 3%-6% of the total costs of inpatient services in public hospitals; preventing the referral of patients (in hospitals affiliated to the MoHME) to the centers out of the supply chain for the purchase of medicines, supplies, equipment, and laboratory and imaging services. • Supporting the retention of physicians in underserved areas in order to increase people’s access to health services; eliminating informal payments; attracting and preserving the physicians in these areas through incentive payments. • Providing financial supports for patients with special and incurable diseases and also for poor patients. • Improving the quality of hoteling in hospitals affiliated to the MoHME through different interventions such as improving hospital amenities, lodging services, and hospital food services. • Improving the quality of visit services in hospitals affiliated to the MoHME through incentive payments to physicians in order to increase the motivations of the healthcare providers, preserve physicians in the public sector, and increase patient satisfaction. • Increasing the presence of resident physicians in hospitals affiliated to the MoHME through incentive payments to physicians in order to increase people’s access to on-time and 24-hour medical care services. • Providing free services for natural childbirth with the aim of promoting natural delivery.	• Reducing OOP payments • Reducing the percentage of households facing CHE • Increasing the rate of natural delivery • Increasing the responsiveness of the health system
Second	• Improving healthcare services in villages and towns with less than 20 thousand inhabitants through recruiting physicians, midwives, and other human resources needed to provide services in rural health centers in underprivileged areas, and adding new drugs to previous list of drugs. • Providing PHC services for a population of over 8.5 to 10 million people who live in suburbs. This population previously has had no access to PHC. In suburbs, there should be a physician per 8 to 15 thousand people, a dietitian per 30 to 50 thousand people, and a psychologist and their experts in laboratory sciences. • Continuing family physician pilot program in two provinces of Fars and Mazandaran, Iran. • Extending family physician program to cities with a population of 20 to 50 thousand people. • Implementing the national plan for promoting and developing self-care. • Strengthening and institutionalizing intra-sectoral cooperation in the field of health at the national, provincial, and city leve.	• Providing access to PHC services for all people • Improving health indicators • Improving the quality of services • Providing comprehensive and integrated services • Designing a hierarchy for services and referral system
Third	• Using an updated relative value units for medical services. • Introducing a dedicated hotline (telephone number: 1690) and a website (http://www.1690.ir) to report any violation and disobedience.	• Creating real justice in payments for various medical specialty services • More realistic medical tariffs • Eliminating informal patient payments

Abbreviations: OOP, out-of-pocket; HTP, health transformation plan; PHC, primary healthcare; MoHME, Ministry of Health and Medical Education; CHE, catastrophic health expenditures.

The HTP is financed through the annual budget allocated by the MoHME; it also receives 10.0% of revenue coming from targeted subsidies and 1.0% of value added tax system.^45^

Along with the reforms in the health system, various aspects of the system need to be monitored and evaluated. Timely monitoring and evaluation of reforms can provide evidence required for (re) directing the reforms.^31,33^ There is not much accurate information about the extent and frequency of informal payments in Iran and no study has been conducted yet to assess the impact of the HTP on OOP payments. Given that the HTP has been one of the most important social programs of the government, monitoring and evaluation of the program can provide many lessons for health system policy-makers. As the first official report on hospitalized patients’ OOP payment after the implementation of the HTP in Iran, this study aimed to determine the extent of OOP payments and frequency of informal cash payment to physicians for inpatient services before and after the implementation of the HTP and its influencing factors in Kurdistan province, Iran. In this study OOP payment for inpatient services was defined as all direct payments made by hospitalized patients for healthcare services, drugs, and diagnostics services in and out of hospitals, and also informal cash payments to physicians The findings of this study can provide a feedback to the healthcare policy-makers to assess the HTP and, if necessary, improve its implementation.

## Methods

### Study Design

This research was conducted as a quasi-experimental study. Quasi-experimental studies include a wide range of non-randomized intervention studies. This type of study is conducted when the use of controlled trial is limited due to logistics or ethical issues. The “simulated before and after” design or the “separate sample pretest-posttest design” are among the most common quasi-experimental designs.^47,48^ This study design is used for studying large populations where it is not possible to randomize the intervention between different groups,^49^ and equivalent samples are selected randomly before and after the intervention.^48^ Our study covered three time periods: before the implementation of the HTP, after the implementation of the first phase of the HTP, and after the implementation of the third phase of the HTP.

### Study Population

The population of this study included all patients discharged from all hospitals across Kurdistan province (including 12 hospitals affiliated to the MoHME, 2 hospitals affiliated to the SSO, and 1 private hospital) during the three time periods: December 22, 2013 to March 20, 2014 (before the implementation of the HTP), 3 months after the implementation of the first phase (May 22, 2014 to August 21, 2014), and 3 months after the implementation of the third phase of the HTP (October 23, 2014 to January 20, 2015).

### Sample Size and Sampling Methods

Considering α = .05 (type 1 error), d = 0.04 (minimal detectable deference) and p = 11.2% (the percentage of informal payments to physicians for inpatient services) and using the equation (1), the target sample size of this study was calculated to be 265 patients.

(1) $$n=\frac{Z^{2}{}_{1-\frac{a}{2}\times P(1-P)}}{d^{2}}$$

As there was the risk of low response rate and to perform a one-time sampling to prevent subsequent errors, the sample size was increased to 300 patients. Sampling was done 3 times (before the first phase of the HTP, after the first phase of the HTP, and after the third phase of the HTP) using multi-stage sampling by hospital type. First, considering the ownership of the hospitals in Kurdistan province, they were classified into three strata, each affiliated to private sector, SSO, and the MoHME, respectively. Then, one hospital affiliated to the private sector, one hospital affiliated to SSO, and four hospitals affiliated to the MoHME were randomly selected. In the second stage, the sample size was calculated in proportion to the number of patients discharged of each hospital. A total of 300 patients were selected via a systematic sampling method. Accordingly, first we used the hospital health information system to calculate the total number of patients discharged from each of the three groups of hospitals during the desired time periods; in proportion to the calculated numbers, the share of each group of hospitals (from a total of 300 samples) was calculated. Then, to calculate the constant interval, we divided the total number of patients in each group of hospitals by the number calculated in the previous step. Subsequently, a starting point was selected randomly and subsequent samples were selected based on constant interval. Data collection was started from the first person on the list and it continued until the time a total of 265 patients were contacted and interviewed via phone calls. In order to reach the target sample size (265 patients) – as some persons on the list did not answer the phone calls – a total of 294, 297, and 296 patients were contacted (from a list of 300 people) for the first, second, and third phases, respectively. In the first and second periods of sampling, before and after the first phase of implementation of the HTP, the hospitals affiliated to the private sector and SSO were selected as the control groups and the hospitals affiliated to the MoHME were selected as the cases, because the first phase of the HTP had made no intervention for the hospitals affiliated to the private sector and SSO.

### Research Instruments and Data Collection

Data about the patients’ demographic characteristics, type of health insurance, hospital stay, and physician expertise, as well as data on the total cost of hospitalization (classified into insurance share, health subsidies share, and OOP patients share) were extracted from a form registered in the HIS. Moreover, the other data about the patients’ location and education, informal payments to physicians, other expenses to buy medicine, supplies, equipment, laboratory tests, and imaging services outside the supply chain were collected through a questionnaire using telephone interviews with patients or their parents (for the patients younger than 18 years of age). In this study, the required data was only collected from patients who were discharged from the hospital; however, because of patient’s conditions as well as cultural context of Iran, there was a low chance of answering the questions by the patients and their families. Thus, in order to communicate better and freely with the patients and their families, answer their questions about the questionnaire, help them write their responses if they were illiterate, and reduce any inconveniences, data collection was performed via telephone interviews (as used in many other similar international studies). If a patient did not cooperate or did not answer after three calls, he/she was randomly replaced with another patient from the list. The validity and reliability of the questionnaire was confirmed in a study by a previous study.^15^ Phone number of the patients were obtained from the hospitals’ HIS system. The samples were selected in February 2015 and their data registered on all the three desired periods were extracted from the hospital HIS systems; in addition, phone calls were made during the same month.

### Statistical Analysis

Using frequency, percentage, and mean, the data was described. Fisher exact test was used to compare the difference in the percentage of informal payments to physicians before and after the implementation of the HTP. Multiple logistic regression was used was used to simultaneously evaluate the relationship between informal payment and independent variables and control the contextual variables. Taking into account P removal = 0.1, backward elimination method was used to build the regression model. In addition, Independent sample *t* test was used to evaluate the difference in mean OOP payments by patients before and after the implementation of the HTP. Data were analyzed using SPSS 20 software. The significance level was set at .05.

## Results

The response rate in the first, second, and third phase of sampling, was 90.1% (265 out of 294 people), 89.2% (265 out of 297 people), and 89.5% (265 out of 296 people) respectively. The mean age (standard deviation, SD) of the studied people at each phase of the study, respectively, was 44.9 (40) years, 42.5 (38) years, and 41.6 (38) years. Table 2 shows the demographic characteristics of the patients studied at different phases of the study.

**Table 2:** Demographic Characteristics of the Study Population

**Variables**	**Phase of the Study**		
	** Before the HTP ** ** No. (%) **	** After the First Phase of the HTP ** ** No. (%) **	** After the Third Phase of the HTP ** ** No. (%) **
Gender			
Male	117 (44.2)	112 (42.3)	115 (43.4)
Female	148 (55.8)	153 (57.7)	150 (56.6)
Level of education			
Illiterate	71 (26.8)	52 (19.6)	41 (15.5)
Low literacy	114 (43.0)	117 (44.2)	115 (43.4)
Average	57 (21.5)	65 (24.5)	71 (26.8)
University	23 (8.7)	31 (11.7)	38 (14.3)
Basic insurance status			
Yes	259 (97.3)	265 (100.0)	265 (100.0)
No	6 (2.3)	0 (0.0)	0 (0.0)
Mean length of stay (SD)	3.7 (2.8) days	3.8 (2.9) days	3.8 (2.5) days

Abbreviations: HTP, health transformation plan; SD, standard deviation.

### Informal Payments to Physician

The first phase of the HTP was implemented only in the hospitals affiliated to the MoHME. At this phase, hospitals not affiliated to the MoHME (private and social security hospitals) were considered as the control groups. The percentages of informal cash payments to physicians before and after the implementation of the first phase of the HTP, were 12.5% and 10.0% in private hospitals, 8.1% and 7.1% in social security hospitals, and 4.5% and 0.0% in the hospitals affiliated to the MoHME respectively. Fisher exact test was used to compare the difference in the percentage of informal cash payments to physician before the first phase and after the third phase of the HTP. In comparison with the time before the implementation of the HTP, after the first phase the percentage of informal cash payments to physicians was significantly reduced only in the hospitals affiliated to the MoHME (*P* = .000). However, the third phase of the HTP was implemented in all the healthcare centers in the country. After the initiation of this phase, no informal cash payment to physicians was reported. In comparison with the time before the HTP, after the third phase the percentage of informal cash payments to physicians was significantly reduced in all the studied hospitals (*P* = .000; Table 3).

**Table 3:** The Frequency and Percentage of Informal Payments to Physicians Based on Type of Hospital in Different Periods of the study

**Type of Hospital**	**Informal Payment**	**Period of the study**				***P*** ** Value** ^a^
		**Before the HTP**	**After the First Phase of HTP**	***P*** ** Value** ^a^	**After the Third Phase of HTP**	
Private	Yes (%)	1 (12.5)	1 (10.0)	1.000	0 (0.0)	1.000
	No (%)	7 (87.5)	9 (90.0)		7 (100.0)	
Social security	Yes (%)	3 (8.1)	3 (7.1)	1.000	0 (0.0)	.093
	No (%)	34 (91.9)	39 (92.9)		43 (100.0)	
Public	Yes (%)	10 (4.5)	0 (0.0)	.000	0 (0.0)	.000
	No (%)	210 (95.5)	213 (100.0)		215 (100.0)	
Total	Yes (%)	14 (5.3)	4 (1.5)	.022	0 (0.0)	.000
	No (%)	251 (94.7)	261 (98.5)		265 (100.0)	

Abbreviation: HTP, health transformation plan. ^a^Fisher exact test.

The results showed that regardless of the period of the study, 94.4% of inpatients (17 out of 18 cases) who practiced informal payments to physicians underwent a surgery regardless of the type of hospitals, 10.7% of those undergoing surgery before the HTP had practiced informal payments to physicians. This was reduced to 3.5% after the implementation of the first phase of the HTP. Fisher exact Test was used to compare the difference in the percentage of informal cash payments to physician before the first phase and after the third phase of the HTP, based on studied variables (Table 4).

**Table 4:** The Frequency and Percentage of Informal Cash Payment to Physicians Based on Study Variables

**Variables**	**Period of the Study**					
	**Before the HTP**		**After the First Phase of HTP**		**After The Third Phase of HTP**	
	**Informal Payment**		**Informal Payment**		**Informal Payment**	
	**Yes (%)**	**No (%)**	**Yes (%)**	**No (%)**	**Yes (%)**	**No (%)**
Gender						
Male^a^	9 (7.7)	108 (92.3)	3 (2.7)	109 (97.3)	0.0	150 (100.0)
Female^a^	5 (3.4)	143 (96.6)	1 (0.7)	152 (99.3)	0.0	115 (100.0)
Type of hospital						
Private	1 (12.5)	7 (87.5)	1 (10.0)	9 (90.0)	0.0	7 (100.0)
Social security	3 (8.1)	34 (91.9)	3 (7.1)	39 (92.9)	0.0	43 (100.0)
Affiliated to MoHME^a^	10 (4.5)	210 (95.5)	0 (0.0)	213 (100.0)	0.0	215 (100.0)
Location						
Urban^a^	5 (2.8)	175 (97.2)	3 (1.4)	206 (98.6)	0.0	204 (100.0)
Rural^a^	9 (10.2)	76 (89.8)	1 (1.8)	55 (98.2)	0.0	61 (100.0)
Type of basic insurance						
Medical services^a^	9 (6.3)	135 (93.7)	2 (1.4)	138 (98.6)	0.0	129 (100.0)
Social scurity	2 (2.2)	90 (97.8)	2 (2.1)	92 (97.9)	0.0	108 (100.0)
Other	3 (13.0)	20 (87.0)	0 (0.0)	31 (100.0)	0.0	28 (100.0)
Supplementary insurance						
Have	1 (5.3)	18 (94.7)	1 (4.5)	21 (95.5)	0.0	21 (100.0)
Not have^a^	13 (5.3)	233 (94.7)	3 (1.2)	240 (98.8)	0.0	244 (100.0)
Household income^b^						
<10 million Rial^a^	12 (8.4)	131 (91.6)	3 (1.9)	158 (98.1)	0.0	207 (100.0)
10-30 million Rial	1 (1.0)	101 (99.0)	0 (0.0)	92 (100.0)	0.0	53 (100.0)
>30 million Rial	1 (5.0)	19 (95.0)	1 (8.3)	11 (91.7)	0.0	5 (100.0)
Specialty physician						
Obstetrics and gynecology	3 (4.2)	68 (95.8)	1 (1.4)	70 (98.6)	0.0	76 (100.0)
General surgery	1 (5.0)	19 (95.0)	0 (0.0)	31 (100.0)	0.0	37 (100.0)
Pediatrics	0 (0.0)	19 (100.0)	0 (0.0)	21 (100.0)	0.0	19 (100.0)
Urology^a^	5 (21.7)	18 (78.3)	1 (4.0)	24 (96.0)	0.0	16 (100.0)
Orthopaedics	0 (0.0)	15 (100.0)	0 (0.0)	24 (100.0)	0.0	11 (100.0)
Otorhinolaryngology	1 (9.1)	10 (90.9)	1 (11.1)	8 (88.9)	0.0	10 (100.0)
Cardiology and pulmonology	0 (0.0)	29 (100.0)	0 (0.0)	13 (100.0)	0.0	22 (100.0)
Ophthalmology	4 (17.4)	19 (82.6)	1 (7.7)	12 (92.8)	0.0	20 (100.0)
Other	0 (0.0)	54 (100.0)	0 (0.0)	58 (100.0)	0.0	54 (100.0)
Type of treatment						
With a surgery^a^	13 (10.6)	109 (89.4)	4 (3.5)	111 (96.5)	0.0	131 (100.0)
Without a surgery	1 (0.7)	142 (99.3)	0 (0.0)	150 (100.0)	0.0	134 (100.0)
Length of stay						
1-2 day^a^	9 (8.7)	94 (71.3)	3 (2.7)	107 (97.3)	0.0	84 (100.0)
3-4 day	3 (3.1)	95 (96.9)	1 (1.2)	82 (98.8)	0.0	116 (100.0)
>5 day	2 (3.1)	62 (96.9)	0 (0.0)	72 (100.0)	0.0	65 (100.0)

Abbreviations: HTP, health transformation plan; MoHME, Ministry of Health and Medical Education. ^a^ *P* value < .05, Results of Fisher exact test. ^b^ Exchange rate: 33810 Iranian Rial to US$1.0.

###  Frequency of Patients Who Referred to out of the Hospital

The results showed that of all patients hospitalized in hospitals affiliated to the MoHME, SSO, and private sector, respectively, 43.6%, 29.7%, and 25.0% before the implementation of the HTP, 2.3%, 21.4%, and 0.0% after the first phase, and 1.9%, 20.9%, and 0.0% after the third phase were referred at least once to the centers out of the hospital supply chain to purchase medicines, supplies, and equipments, and receive laboratory and imaging service (Table 5).

**Table 5:** The Frequency and Percentage of Patients Who Referred to Out of the Hospital Supply Chain Based on Type of Hospital in Different Periods of the Study

**Type of Hospital**	**Referred to the Supply Chain of Hospital**	**Period of the Study**				***P*** ** Value** ^a^
		**Before the HTP**	**After the First Phase of HTP**	***P*** ** Value** ^a^	**After the Third Phase of HTP**	
Private	Yes (%)	2 (25.0)	0 (0.0)	.183	0 (0.0)	.466
	No (%)	6 (75.0)	10 (100.0)		7 (100.0)	
Social security	Yes (%)	11 (29.7)	8 (21.4)	.301	9 (20.9)	.441
	No (%)	26 (70.3)	34 (78.6)		34 (79.1)	
Public	Yes (%)	96 (43.6)	5 (2.3)	.000	4 (1.9)	.000
	No (%)	124 (56.4)	208 (97.7)		211 (98.1)	

Abbreviation: HTP, health transformation plan. ^a^ Fisher exact test.

###  Factors Effecting Informal Payments to Physician

Multiple logistic regression models were used to assess the simultaneous effects of variables on informal payments to physicians. The percentage of hospitalized patients who had informal payments to physician is the dependent variable. The independent variables were the followings: period of the study, type of hospital, location of residence, type of basic insurance used by the, status of supplementary insurance used by the patient, household income, and type of physician’s specialty (Appendix 1). These variables were entered into multiple logistic regression models and analyzed using the backward elimination method (Table 6). The results showed that the odds ratio (OR) of informal payments to physicians declined after the implementation of the HTP. For example, patients who were hospitalized after the HTP were 70.0% less likely to practice unofficial payment than those hospitalized before it (OR = 0.3, CI = 0.1-1.1).

**Table 6:** Relationship Between Informal Payments to Physicians and the Studied Variables

**Independent Variables**	**OR**	**95% CI**		**Definition of the Variables**
		**Lowest**	**Highest**	
Period of the study				
Before the HTP^a^	‏-	‏-	‏-	Reference category
After the first phase of the HTP	0.3	0.1	1.1	
After the third phase of the HTP	0.1	0.0	0.6	
Type of hospital				
Affiliated to MoHME	‏-	‏-	‏-	Reference category
Not affiliated to MoHME	4.6	1.3	16.0	Including private and social security hospitals
Location				
Urban	‏-	‏-	‏-	Reference category
Rural	3.6	1.1	11.2	
Type of basic insurance				
Medical services	-	-	-	Reference category
Social security	0.7	0.2	2.7	
Other	1.5	0.3	7.1	Including Armed forces, Relief and Welfare Committee, banks
Supplementary insurance				
Not have	-	-	-	Reference category
Have	0.8	0.1	8.8	
Household income				
<10 million Rial	‏-	‏-	‏-	Reference category
10-30 million Rial	0.2	0.0	1.7	
>30 million Rial	2.0	0.2	19.8	
Specialty of the physician				
Obstetrics and gynecology	‏-	‏-	‏-	Reference category
General surgery, orthopaedics	0.6	0.1	6.6	
Ophthalmology, otorhinolaryngology, urology	4.3	1.1	16.5	
Other	0.2	0.0	2.5	Including pediatrics, cardiology, pulmonology, gastroenterology, endocrinology, hematology, oncology, internal medicine, infectious disease, neurology

Abbreviations: HTP, health transformation plan; MoHME, Ministry of Health and Medical Education. ^a^ All the variables were compared with the Reference category; 2LL = 90.7a; Nagelkerke R square = 0.5.

Those discharged from the private sector and social security hospitals were about 4.5 times more likely to pay unofficially to physician than those discharged from hospitals affiliated to the MoHME (OR = 4.6, CI = 1.3-16.0). In addition, the OR of informal payments to physicians for people living in rural areas was almost 3.5 times higher than those living in urban settings (OR = 3.6, CI = 1.1-16.0).

Type of basic insurance scheme had an impact on the OR of informal payments to physicians. Compared with those under the coverage of medical services insurance, patients under the coverage of social security insurance and those under the coverage of other types of insurance, respectively, were more and less likely to practice informal payments to physicians. Moreover, patients with supplementary insurance were more prone to informal payments to physicians than those without supplementary insurance (OR = 0.8, CI = 0.1-8.8).

Patients living in the households with a monthly income of more than 30 million Rial (equal to US$850) were about two times more prone to informal payments than those with a monthly income of less than 10 million Rial (equal to US$280) (OR = 2.0, CI = 0.2-19.8). In addition, patients living in the households with monthly income of 10 to 20 Rial were less likely to pay unofficially than those with a monthly income of less than 10 million Rial (OR = 0.2, CI = 0.0-1.7).

Hospitalized patients who received services form nephrologists, ophthalmologist, urologists, and otolaryngologists were 4.2 times more prone to informal payments to physicians compared to other patients receiving gynecology services (OR = 4.3, CI = 1.1-16.5).

###  Cost of Inpatient Hospitalization and Out-of-Pocket Payment

Table 7 presents the extent and percentage of hospital costs by types of cost, hospital, and time period.

**Table 7:** The Cost of Inpatient OOP Payments by Type of Hospital and Period of the Study

**Variables**	**Period of the study**																							
	**After the third phase of the HTP**								**After the first phase of the HTP**								**Before the HTP**							
	**Type of hospital**								**Type of hospital**								**Type of hospital**							
	**Private**			**Social Security**			**Affiliated to MoHME**			**Private**			**Social Security**			**Affiliated to MoHME**			**Private**		**Social Security**		**Affiliated to MoHME**	
Cost per inpatient^b^	*P* ^a^	SD	Mean (%)	*P* ^a^	SD	Mean (%)	*P* ^a^	SD	Mean (%)	*P* ^a^	SD	Mean (%)	*P* ^a^	SD	Mean (%)	*P* ^a^	SD	Mean (%)	SD	Mean (%)	SD	Mean (%)	SD	Mean (%)
Formal OOP to hospital	0.462	166.0	66.4 (6.5)	0.022	12.5	4.4 (1.2)	.000	18.2	14.0 (3.3)	.991	188.1	129.6 (24.6)	0.861	41.3	17.7 (5.7)	.000	18.6	17.3 (5.7)	150.4	128.9 (25.6)	37.9	19.2 (7.7)	45.3	33.5 (13.6)
Formal payment out of hospital	0.314	0	0	0.251	8.2	9.3 (2.4)	.000	2.3	0.3 (0.1)	.311	0	0	0.612	16.2	11.5 (3.7)	.000	1.9	0.3 (0.1)	24.9	9.6 (1.9)	22.4	13.8 (5.5)	57.8	19.9 (8.1)
Informal payment to physician	0.352	0	0	0.131	0	0	.000	0	0	.480	187.1	59.1 (11.2)	0.682	17.5	4.4 (1.4)	.000	0	0	41.8	14.8 (3.0)	26.0	6.5 (2.6)	33.0	6.0 (2.3)
**Total OOP**	0.223	166.0	66.4 (6.5)	0.022	14.7	13.7 (3.6)	.000	18.9	14.3 (3.4)	.652	239.2	188.7 (35.8)	0.644	45.7	33.7 (10.8)	.000	18.5	17.6 (5.8)	48.4	153.3 (30.5)	63.9	39.6 (15.8)	80.8	59.4 (24.0)
Share of the HTP fund	-	0	0	-	0	0	.000	53.4	36.3 (8.4)	-	0	0	-	0	0	.000	55.2	25.6 (8.5)	0	0	0	0	0	0
Contribution of insurance^c^	0.001	204.1	959.9 (93.5)	0.061	527.1	368.0 (96.4)	.000	428.2	379.6 (88.2)	.632	263	338.9 (64.2)	0.261	338.3	279.3 (89.2)	.021	389.4	260.1 (85.7)	279.5	349.1 (69.5)	156.6	211.5 (84.2)	240.2	187.5 (76.0)
**Total cost**	0.001	230.8	959.9 (100)	0.021	185.6	381.6 (100)	.000	485.1	430.2 (100)	.841	336.3	527.6 (100)	0.402	348.3	313.0 (100)	.000	418.3	336.8 (100)	194.5	502.4 (100)	300.1	251.1 (100)	269.1	246.9 (100)

Abbreviations: SD, standard deviation; HTP, health transformation plan; MoHME, Ministry of Health and Medical Education; OOP, Out-of-Pocket. ^a^ Results of independent samples *t* test. ^b^ Exchange rate: 33 810 Iranian Rial to US$ 1.0. ^c^ Both of basic and supplementary insurance.

The mean (SD) OOP payment per every patient hospitalized in the hospitals affiliated to the MoHME before the implementation of the HTP, and after the first and third phases of the HTP, respectively, was US$59.4 (80.8), US$17.6 (18.5), and US$14.3 (18.9). There were significant differences between the mean OOP payment before the implementation of the first phase with that after the first phase of the HTP (*P* = .000) and after the third phase of the HTP (*P* = .000).

In addition, the mean (SD) OOP payment per every patient hospitalized in hospital affiliated to the SSO before the implementation of the HTP, and after the first third phases of the HTP, respectively, was US$39.6 (63.9), US$33.7 (45.7), and US$13.7 (14.7). There were no significant differences between the mean OOP payment before the implementation of the first phase with that after the first phase of the HTP (*P* = .644), however it was significantly different from the time after the third phase of the HTP (*P* = .022).

The mean (SD) OOP payment per every patient hospitalized in the private hospital before the implementation of the HTP, and after the first and third phases of the HTP, respectively, was US$153.3 (48.8), US$188.7 (239.2), and US$66.4 (166.0). There were no significant differences between the mean OOP payment before the implementation of the first phase with that after the first phase of the HTP (*P* = .652) and after the third phase of the HTP (*P* = .223).

The mean (SD) informal cash payments to physicians per every patient hospitalized in hospitals affiliated to the MoHME before the HTP was US$6.0 (33.0) which was reduced to zero after the implementation of the HTP. There was a significant difference between mean informal payments to physicians per every patient before and after the implementation of the HTP (*P* = .000).

Moreover, the mean (SD) informal cash payments to physicians per every patient hospitalized in the hospital affiliated to SSO before the implementation of the HTP, and after the first and third phases of the HTP, respectively, was US$6.5 (26.0), US$4.4 (17.5), and US$0 (0.0). There were no significant differences between the mean informal payments to physicians before the implementation of the first phase with that after the first phase of the HTP (*P* = .682) and after the third phase of the HTP (*P* = .131).

The mean (SD) informal cash payments to physicians per every patient hospitalized in private hospital before the implementation of the HTP, and after the first and third phases of the HTP, respectively, was US$14.8 (41.8), US$59.1 (187.1), and US$0.0 (0.0) There were no significant differences between the mean informal payments to physicians before the implementation of the first phase with that after the first phase of the HTP (*P* = .480) and after the third phase of the HTP (*P* = .352).

The mean percentages of OOP payments in hospitals affiliated to the MoHME, SSO, and private sector, respectively, were 24.0%, 15.8%, and 30.5% before the HTP while they become 5.8%, 10.8%, and 35.8% after the first phase, after the third phase of the HTP, the OOP payments of patients hospitalized in hospitals affiliated to the MoHME, SSO, and the private sector accounted for 3.4%, 3.6%, and 6.5% of the total cost of hospitalization.

Of all patients hospitalized in hospitals affiliated to the MoHME, SSO, and private sector, respectively, 43.6%, 29.4%, and 25.0% before the implementation of the HTP, 2.3%, 22.0%, and 0.0% after the first phase, and 2.0%, 20.0%, and 0.0% after the third phase were referred at least once to the centers out of the hospital supply chain to purchase medicines, supplies, and equipments, and receive laboratory and imaging service. In hospitals affiliated to MoHME, there was a significant difference between out-of-hospital OOP payments before the HTP and after the HTP.

## Discussion

The mean (SD) OOP payments before the HTP and after the third phase, respectively, were US$59.4 (80.8) and US$14.3 (3.4) (*P* = .00) in hospitals affiliated to the MoHME, US$39.6 (63.9) and US$13.7 (14.7) (*P* = .02) in SSO hospitals, and US$153.3 (48.4) and US$66.4 (166.0) (*P* = .22) in private sector hospitals. The reduction of OOP can be attributed to different factors including increased coverage of inpatient expenditures by health insurances schemes, reduction of informal payments, providing free-of-charge natural childbirth services in hospitals affiliated to the MoHME, utilization of the health transformation fund for paying part of hospitalization costs, and not referring patients hospitalized in the hospitals affiliated to the MoHME to purchase medicines, medical supplies, and diagnostic services out of the supply chain.^44-46^ The results of a study in Iran showed that the mean OOP payment of mothers who had a delivery in university hospitals was significantly reduced after HTP, as compared with the time before the HTP. According to the mentioned study, the mean OOP payment was US$29.5 before implementing the HTP which decreased to US$9.9 after the first phase and to US$10.2 after the third phase of the HTP.^50^

The high levels of OOP payments can be economically catastrophic for the poor.^3,45^ In recent years and before the implementation of the HTP, a high proportion of households has been exposed to CHE in Iran.^3,13,37,38^ This happened because the insurance organizations failed to cover the costs, therefore, a large amount of the expenses for health services were compensated through OOP.^2^ The implementation of HTP reduced the volume of direct expenditures on health services in Turkey. Accordingly, the share of health spending as a proportion of non-food costs changed from 3.1% in 2003 to 2.4% in 2011. In addition, the percentage of households faced with CHE was decreased by three times during the same period.^36^

Our findings also showed that unlike the SSO and private sector hospitals, after the first phase of the HTP the percentage of informal cash payments to physicians was significantly reduced in hospitals affiliated to the MoHME (*P* = .00). The percentage of informal payments to physicians in hospitals affiliated to the MoHME, Social Security and private sector, was 4.5%, 8.1%, and 12.5% respectively, before the implementation of the first phase of the HTP and 0.0%, 7.1%, and 10.0% after the implementation of the first phase of the HTP. The first phase of the HTP was performed only in hospitals affiliated to the MoHME (except for covering people without basic health insurance). After the implementation of the third phase, no informal payment to physicians was found in the studied cases. During the third phase of the HTP, the book of the updated relative value units (RVU) of health services was issued and it was applied to all health centers across the country. Under this phase, the tariffs of most services increased dramatically. For example, the tariff of vaginal delivery changed from 15K (before the HTP) to 50K (after the implementation of the third phase).^50^ Most physicians had complained about unrealistic tariffs system in the course of recent years, considering it as the main reason for receiving informal payments.^21,22^ In 2013, the percentage of informal payments to physicians was 2.0% in hospitals affiliated to the MoHME, 15.0% in private hospitals, 34.0% in Social Security hospitals, and 11.3% in all types of hospital.^15^

One study in Greece showed that in 2013, 74.4% of mothers who had used the childbirth services in the public sector were forced to practice informal payment.^51^ According to a survey conducted in Bulgaria in 2010, informal payments were reported even 10 years after the implementation of formal co-payments for services in basic benefits package, which were designed to reduce unofficial payments. About 13.0% of the users of outpatient care and 33.0% of the users of inpatient care had reported unofficial payments.^26^ The results of a study in Serbia showed that 5.7% of health service users experienced informal payments.^52^

In our study, the proportion of informal payments to physicians, which was defined as a percentage of hospitalized patients’ OOP, was 10.1%, 16.4%, and 9.6% respectively in the hospitals affiliated to the MoHME, Social Security, and private sector before the implementation of the HTP. The rate changed to 0.0%, 13.0%, and 31.1%, respectively, after the implementation of the first phase of the HTP. This percentage dropped to zero and there were no reports of informal payments to physicians in the studied hospitals after the implementation of the third phase of the HTP. The estimates indicate that the informal payments in low-income countries account for 10.0% to 45.0% of the total OOP for health.^25^ A study conducted in 2013 in Iran (Urmia province in northwest of Iran) indicated that informal payments accounted for 15.0% of the total OOP to hospitals.^15^

In our study, after the implementation of the HTP (as compared with the time before its implementation) there was a significant reduction in the number of patients referred to hospitals outside the MOHME hospital supply chain. In addition, the share of OOP payments out of hospital from the inpatient total OOP payments was significantly decreased. The observed share was 36% in MOHME hospitals before the implementation of the HTP which reduced to 2% after the implementation of the third phase of the HTP. Prior to the implementation of the HTP, patients admitted to the hospitals affiliated to the MoHME were occasionally referred to the centers out of the hospital to buy some medical supplies. Since these expenses were not recorded in hospital bills, they were not covered by basic health insurance organizations. Ghiasvand et al conducted a study in public hospitals in Tehran in 2012 and showed that 68.5% of the total OOP payments of patients out of the hospital was for the purchase of medicines, medical equipment, and diagnostic services.^13^ In some studies, requesting patients to provide medical goods and equipment (which indeed must be provided by the hospitals) are considered as a type of informal payment.^52^ The other countries whose healthcare system is in transition have a similar condition.^53^ The payments for “bought and brought healthcare goods” are also observed in other countries.^52^

The extent and percentage of contribution of health insurance organizations in financing inpatient services have increased in all hospitals after the implementation of the HTP. The Iranian National Health Accounts of 2008 showed that the 3 main insurance organizations in Iran, ie, Iranian health insurance, social security, and armed forces insurance organizations, incurred 6.6%, 10.9%, and 1.5% of the total healthcare expenditures respectively, which indicated the inefficiency of insurers in covering the costs of services.^38^

Patients discharged from the hospitals after the initiation of the HTP, compared with those discharged from the hospitals before the HTP plan, had lower odd ratios for informal payments to the physicians, our research revealed.

People discharged from the hospitals affiliated to the MoHME, compared with those discharged from other hospitals, had lower ORs for informal payments to physicians. This might be due to the implementation of the first phase of HTP in the hospitals affiliated to the MoHME, which enhanced the supervision of universities of medical sciences over these hospitals. The extent and percentage of informal payments to physicians in hospitals affiliated to the MoHME have been reported lower than that in other hospitals in Iran.^15^ A Greek study showed that those who were hospitalized in private hospitals were 2.1 times more prone to pay bonuses to nurses than those hospitalized in public hospitals.^27^ A Turkish study also showed that the type of the ownership of the service provider had a significant relationship with informal payments.^54^

Residential location was one of the factors affecting informal payments to physicians. People living in rural areas were four times more prone to pay informally to physicians than those living in urban settings. A study conducted in Albania showed that the residential location was one of the factors influencing the volume of informal payments.^54^ This was also confirmed in an Iranian study.^15^

Hospitalized patients who received services from nephrologists, ophthalmologist, and otolaryngologists were more prone to informal payments to physicians, compared to patients who visited other specialists. A study in Iran showed that 90.0% of people who had informal payments to physicians were those who had undergone a surgery; moreover, general surgeons received the highest amount of informal payments (42.0%), followed by otolaryngology surgeons and maxillofacial surgeons (24.0%).^15^ A Greek study showed that people undergoing surgery were 137.0% more prone to informal payments than those who did not undergo surgery.^27^

Patients living in the households with a monthly income of 10 to 30 million Rial were less prone to informal payments while those with higher incomes had a higher chance for informal payments to physicians. An increase in average monthly household income may increase the volume of informal payments to physicians.^15^ Results of a study in Albania showed a weak relationship between income and the informal payments; however, it showed a positive relationship between the volume of informal payments and patients’ level of income.^55^

Our study showed that patients under the coverage of social security insurance were less prone to informal payments to physicians, in comparison to patients under the coverage of other insurance schemes. Despite higher percentage of informal payments to physicians in social security hospitals, patients covered by social security insurance were less prone to informal payments to physicians. This is due to the fact that the physicians in the social security hospitals had fewer requests for informal payments from people under the coverage of the social security insurance (3.5%) than those under the coverage of other insurance companies (8.6%). Previous researches showed no correlation between the informal payments and type of insurance.^16,25^ In our study, patients without any medical insurance were more likely to pay unofficially. The percentage of patients with supplementary insurance in the weakest and strongest income level group were 4.5% and 40.5% respectively, however, about 89.0% of people with informal payments to physicians were located in the poorest income groups.

## Limitations

In this study, part of the data was collected through making phone calls. In some cases, due to the lack of access to landline or cell phone number of the patient or the patient’s family members, it was not possible to interview some of the subjects.

In our study, the samples were selected in proportion to the size of the hospitals in different sectors. As private hospitals had a very low share in the total number of patients discharged from hospitals, a very low number of patients were selected from this type of hospital. Accordingly, the results obtained for the private hospital should be interpreted with caution. Also, our collected data are prone to the risk of recall bias and a probability of over- or under-reporting of the costs paid by patients. Moreover, this study was carried out in Kurdistan province, west of Iran; hence, the results may not represent a full picture of formal and informal payments in the whole country.

## Conclusion

OOP has had a rising trend in the course of recent years and has accounted for over 50.0% of the total healthcare costs in Iran. High level of OOP might be due to the low share of the health budget in the public funds, inefficiencies of insurance organizations, receiving extra-tariff payments from patients, and referring patients out of the hospital for the purchase of medicines, equipment, and supplies. Due to the high proportion of OOP (formal and informal) and because of its negative effects on equity and access to health services, policy-makers have focused on and implemented HTP to reduce such payments. Based on our findings, it seems that the implementation of the HTP, at least in a short-run, has had a noticeable impact on the reduction of OOP expenditures and informal payments to physicians in Kurdistan province as compared with the time before the implementation of the HTP. This observation might have been caused by the transformation plan (as it was intended), however further research is required to assess the causality of the link between the reform the observed reductions in such payments in the short and long term periods.

## Acknowledgements

This paper was extracted from a PhD dissertation in Health Policy. We would like to thank all those who helped us in this study including the staffs working in Kurdistan University of Medical Sciences, Sanandaj, Iran and Social Determinant of Health Research Center, Sanandaj, Iran who helped us in collecting the data. This research was funded by the Tehran University of Medical Sciences, Tehran, Iran (No. 9121557002) and Kurdistan University of Medical Sciences, Sanandaj, Iran (No. 1394.301).

## Ethical issues

This study was approved by the ethics committee of Tehran University of Medical Sciences, Tehran, Iran (Approval No. IR.TUMS.REC.1394.1573).

## Competing interests

The authors declare they have no competing interests.

## Authors’ contributions

Study topic (AR, BP, and GM); Study methodology (AR, BP, GM, and AT); Data collection (BP, GM, HG, and TG); Carried out data analysis (AR, BP, and GM); Contribute to the writing and several editing of the manuscript (All authors).

## Authors’ affiliations

^1^Department of Health Services Management and Economics, School of Public Health, Tehran University of Medical Sciences, Tehran, Iran. ^2^Knowledge Utilization Research Center, Tehran University of Medical Sciences, Tehran, Iran. ^3^Social Determinants of Health Research Center, Kurdistan University of Medical Sciences, Sanandaj, Iran. ^4^Department of Global Health and Public Policy, School of Public Health, Tehran University of Medical Sciences, Tehran, Iran. ^5^National Academy of Medical Sciences, Tehran, Iran. ^6^College of Health and Life Sciences, Brunel University London, London, UK. ^7^Deputy of Treatment, Kurdistan University of Medical Sciences, Sanandaj, Iran. ^8^Department of Surgery, Faculty of Medicine, Kurdistan University of Medical Sciences, Sanandaj, Iran.

## Key messages

**Implications for policy makers**

- It seems that the implementation of the health transformation plan (HTP) reduced the mean out-of-pocket (OOP) payments for inpatient services in Kurdistan province, Iran.
- After the implementation of the third phase of the HTP, during the study period, we did not found any informal cash payments to physicians for inpatient services.
- Several factors significantly increased the probability of informal payments to physician including the followings: being discharged from private sector and social security hospitals, being discharged from hospitals before the implementation of the HTP, living in rural areas, being visited by an ophthalmologist, urologist or otolaryngologists.
- Health policy-makers should pay attention to patients’ payments for “bought and brought health care goods” purchased from out-of-hospital supply chain.

**Implications for public**

After the implementation of the health transformation plan (HTP), the amount of out-of-pocket (OOP) payments for inpatient services was reduced. Reduction of OOP payments for inpatient services improves people’s access to such services and enhances the equity. After the implementation of the third phase of the HTP no informal cash payments to physician was reported. The elimination of informal payments reduces the probability of corruption in the health system.

## Appendix 1

**Appendix 1.** Descriptive Statistics of All the Variables Used in the Regression Model

**Variables**		**Have Informal Payment**	**Number**
	**No (%)**	**Yes (%)**	
Period of the study			
Before the HTP	251 (94.7)	14 (5.3)	265
After the first phase of HTP	261 (98.5)	4 (1.5)	265
After the third phase of HTP	265 (100.0)	0 (0.0)	265
Type of hospital			
Affiliated to MoHME	638 (98.5)	10 (1.5)	648
Not affiliated to MoHME	139 (94.6)	8 (5.4)	147
Location			
Urban	585 (98.7)	8 (1.3)	593
Rural	192 (95.1)	10 (4.9)	202
Type of basic insurance			
Medical services	402 (97.3)	11 (2.7)	413
Social security	209 (98.6)	4 (1.4)	294
Armed forces	33 (100.0)	0 (0.0)	33
Relief and Welfare committee	42 (93.3)	3 (6.7)	45
Banks	4 (100.0)	0 (0.0)	4
Supplementary insurance			
Not have	717 (97.8)	16 (2.2)	733
Have	60 (96.8)	2 (3.2)	62
Household income			
<10 million Rial	496 (97.1)	15 (2.9)	511
10-30 million Rial	246 (99.6)	1 (0.4)	247
>30 million Rial	35 (94.6)	2 (5.4)	37
Specialty of the physician			
Obstetrics and Gynecology	214 (98.2)	4 (1.8)	218
General surgery	87 (98.9)	1 (1.1)	88
Orthopaedic	50 (100.0)	0 (0.0)	50
Ophthalmology	51 (91.1)	5 (8.9)	56
Otorhinolaryngology	28 (93.3)	2 (6.7)	30
Urology	58 (90.6)	6 (9.4)	64
Pediatrics	59 (100.0)	0 (0.0)	59
Cardiology	43 (100.0)	0 (0.0)	43
Internal medicine	63 (100.0)	0 (0.0)	63
Pulmonology	21 (100.0)	0 (0.0)	21
Gastroenterology	13 (100.0)	0 (0.0)	13
Endocrinology	4 (100.0)	0 (0.0)	4
Hematology	6 (100.0)	0 (0.0)	6
Oncology	10 (100.0)	0 (0.0)	10
Neurology	0 (100.0)	0 (0.0)	45
Infectious disease	0 (100.0)	0 (0.0)	25
Type of treatment			
With a surgery*	351 (95.4)	17 (4.6)	368
Without a surgery	426 (99.8)	1 (0.2)	427
Length of stay			
1-2 day*	285 (96.0)	12 (4.0)	297
3-4 day	293 (98.7)	4 (1.3)	297
>5 day	199 (99.0)	2 (1.0)	201

Abbreviation: HTP, health transformation plan‏.
